# Impact of Self-Rated Health on Progression to a Metabolically Unhealthy Phenotype in Metabolically Healthy Obese and Non-Obese Individuals

**DOI:** 10.3390/jcm8010034

**Published:** 2019-01-01

**Authors:** Mi-Hyun Kim, Yoosoo Chang, Hyun-Suk Jung, Hocheol Shin, Seungho Ryu

**Affiliations:** 1 Centre for Cohort Studies, Total Healthcare Centre, Kangbuk Samsung Hospital, Sungkyunkwan University School of Medicine, Seoul 04514, Korea; mh0303.kim@samsung.com (M.-H.K.); hs1601.jung@samsung.com (H.-S.J.); hcfm.shin@samsung.com (H.S.); 2 Department of Occupational and Environmental Medicine, Kangbuk Samsung Hospital, Sungkyunkwan University School of Medicine, Seoul 03181, Korea; 3 Department of Clinical Research Design and Evaluation, SAIHST, Sungkyunkwan University, Seoul 06351, Korea; 4 Department of Family Medicine, Kangbuk Samsung Hospital, Sungkyunkwan University School of Medicine, Seoul 03181, Korea

**Keywords:** body mass index, self-rated health, metabolic health, cohort study, obesity

## Abstract

We examined the association between self-rated health (SRH), a subjective measure of an individual’s health status, and the incidence of metabolic abnormalities, as well as the effect of obesity on this association in metabolically healthy individuals. The cohort study included 85,377 metabolically healthy men and women who were followed annually or biennially for a median of 3.0 years (interquartile range, 1.9–4.1 years). A parametric proportional hazard model was used to assess hazard ratios (HRs) and 95% confidence intervals (CIs) for the association between SRH and the incidence of metabolic abnormalities. During 258,689.03 person-years, 40,858 participants developed metabolic abnormalities. Poorer SRH was significantly associated with increased risk of developing any metabolic abnormality including hypertriglyceridemia, high homeostasis model assessment of insulin resistance (HOMA-IR), and fatty liver in a dose-dependent manner (*p* for trend <0.05). The association between SRH and progression to metabolically unhealthy status was much stronger in individuals with obesity than those without, especially in relation to any metabolic abnormality, fatty liver, and high C-reactive protein (all *p* for interaction by obesity <0.05). The multivariable-adjusted HR (95% CI) for any metabolic abnormality comparing the “poor or very poor” vs. the “very good” self-rated health category was 0.97 (0.90–1.05) among non-obese subjects, whereas the corresponding HR (95% CI) among obese subjects was 1.25 (1.02–1.52). Low SRH, as assessed by a single question, was independently associated with increased risk of progression to metabolically unhealthy status in metabolically healthy individuals, especially metabolically healthy individuals with obesity. SRH may help identify individuals at high risk for progression to metabolically unhealthy status.

## 1. Introduction

Self-rated health (SRH) is a simple measure of assessing an individual’s subjective perception of their own health status by a single question, “In general, how would you rate your health?” SRH reflects both psychological and physical health status and it is one of the most widely used measures for general health status in clinical settings, public research, and epidemiological research [1]. SRH has been extensively studied and consistently associated with a wide range of diseases and conditions including cardiovascular disease (CVD), type 2 diabetes mellitus (T2D), stroke, lung disease, arthritis, depression, functional impairment, postoperative outcome, and mortality [2,3,4,5]. SRH appears to be an independent predictor of mortality after controlling for sociodemographic variables, behavioural and psychosocial factors, and various aspects of health status, indicating that SRH may be a very inclusive measure of health reflecting health aspects relevant to survival that are not covered by other health indicators [6].

During the past few decades, the prevalence of obesity has dramatically increased and has become a major public health issue worldwide [7]. However, a proportion of subjects with obesity appear to have a favourable metabolic profile and better prognosis [8,9,10,11]; this group is referred to as metabolically healthy individuals with obesity. On the other hand, there are individuals who despite having normal weight, have obesity-related metabolic abnormalities [12]. Being metabolically unhealthy also increases the risk of developing CVD and T2D regardless of body mass index (BMI) status [13,14]. Indeed, for a certain BMI, the risk of cardio-metabolic disease and death can vary substantially among subjects according to different metabolic phenotypes [12]. Currently, the risk factors of progression from a healthy state to an unhealthy state have not been well characterised. We hypothesised that SRH, an inclusive measure of health status, could affect the incidence of metabolic abnormalities in a metabolically healthy population. No previous studies have evaluated the longitudinal association between SRH and the incidence of metabolic abnormalities.

We evaluated the association between SRH and incidence of metabolic abnormalities in a large cohort of young and middle-aged Korean men and women who participated in a health screening examination program. We also investigated whether the effect of SRH on incidence of metabolic abnormalities differs by obesity, a strong determinant of metabolic disorders. 

## 2. Method

### 2.1. Study Subjects

This retrospective cohort study used data of the Kangbuk Samsung Health Study (KSHS), a cohort study of Korean men and women who underwent a health examination annually or biennially at the Kangbuk Samsung Hospital Health Screening Centres in Seoul and Suwon, South Korea [15]. The majority of participants (over 80%) were employees of various companies and local governmental organizations or their spouses. In South Korea, the Industrial Safety and Health Law requires annual or biennial health screening examinations of all employees, free of charge. The rest of the examinees voluntarily purchased health screening exams at the healthcare centre.

This study population was restricted to KSHS participants who underwent comprehensive examination and completed SRH questionnaire between 1 January 2011 and 31 December 2016, and who had at least one follow-up visit through 31 December 2017 (*n* = 276,244). For this analysis, we excluded 190,867 participants who had any components of metabolic syndrome at baseline, evidence of liver disease, or factors that could influence metabolic syndrome traits as follows: missing information on SRH and metabolic parameters (*n* = 60,027); a history of malignancies (*n* = 6245); a history of liver cirrhosis (*n* = 88); a history of CVD (*n* = 2733); fatty liver on ultrasonography (*n* = 78,262); homeostasis model assessment of insulin resistance (HOMA-IR) ≥2.5 (*n* = 28,601); fasting glucose ≥100 mg/dL or current use of glucose-lowering agents (*n* = 66,942); blood pressure ≥135/85 mmHg or current use of blood pressure-lowering agents (*n* = 44,411); triglyceride levels ≥150 mg/dL (*n* = 55,127); high-density lipoprotein-cholesterol (HDL-C) <40 mg/dL in men and <50 mg/dL in women (*n* = 36,758); and high-sensitivity C-reactive protein (hsCRP) ≥1.0 mg/L (*n* = 51,484). Some individuals met more than one exclusion criterion, and the total number of metabolically healthy subjects included in the study was 85,377.

This study was approved by the Institutional Review Board of Kangbuk Samsung Hospital (IRB No. KBSMC 2017-08-044), which exempted the requirements for informed consent as we only retrospectively assessed de-identified data that were collected routinely as a part of health screening examination.

### 2.2. Measurements

Baseline and follow-up examinations were conducted at Kangbuk Samsung Hospital Health Screening Centre clinics in Seoul and Suwon. All participants were asked to complete a standardised, self-administered questionnaire about SRH, demographic characteristics, alcohol consumption, smoking status, physical activity, education level, medical history, and medication use. SRH was assessed using the Korean version of a single question in order to measure general health, [16] which was “In general, how would you rate your health?" with the possible choices being “very good” (1), “good” (2), “fair” (3), “poor” (4) or “very poor” (5) [17]. There have been a number of studies investigating the content validity of SRH in populations of various ethnicities and races [16,18].

Health behaviours and education levels were categorised as follows: smoking status (never, former, or current smoker), alcohol consumption (≤20 g/day and >20 g/day), and education level (less than college graduate or college graduate or more). Physical activity levels were assessed using the validated Korean version of the International Physical Activity Questionnaire Short Form [19,20]. Physical activity levels were classified into three categories: inactive, minimally active, and health-enhancing physical activity (HEPA) [20]. HEPA was defined as physical activity that met either of two criteria: (i) vigorous intensity activity on three or more days per week accumulating ≥1500 metabolic equivalent (MET) min/week; or (ii) seven days of any combination of walking, moderate intensity, or vigorous intensity activities achieving at least 3000 MET min/week [21]. Sleep duration was assessed with the Pittsburgh Sleep Quality Index (PSQI) [22], which has also been validated for use in Korea [23]. Sleep duration was categorised as ≤5, 6, 7, 8, or ≥9 h/day [24,25]. Depression was assessed using the Korean version of the Centre for Epidemiologic Studies Depression (CESD) Scale [26,27]. CESD scores were categorised into <16 (normal), 16–<25 (depressive symptoms) and ≥25 (clinical depression). Usual dietary intake was assessed using the Korean version of a 103-item self-administered food frequency questionnaire [28].

Height, weight, and blood pressure were measured by trained nurses. Height was measured to the nearest 0.1 cm using a stadiometer with the examinee standing without shoes. Weight was measured to the nearest 0.1 kg in a light gown while barefoot using a bioimpedance analyser (Inbody 720, Biospace Co., Seoul, Korea). Obesity was defined as a BMI ≥25 kg/m^2^ according to Asian-specific criteria [29]. Waist circumference was measured to the nearest 0.1 cm at the midpoint between the bottom of the rib cage and the top of the iliac crest with the subjects standing, their weight equally distributed on both feet, their arms at their sides, and head facing straight forward. Abdominal obesity was defined as waist circumference ≥90 cm for men and ≥85 cm for women that are specific for Korean populations [30,31]. Blood pressure was measured by trained nurses using an automated oscillometric device (53000, Welch Allyn, New York, NY, USA) while examinees were in a sitting position with the arm supported at heart level. Three readings were recorded and the average of the second and third readings was used in the analysis. Hypertension was defined as a systolic blood pressure ≥140 mmHg, a diastolic blood pressure ≥90 mmHg, or the use of antihypertensive medications.

Blood samples were drawn from the antecubital vein after at least a 10-h fast. Serum biochemical parameters were measured, including glucose, alanine aminotransferase (ALT), insulin, total cholesterol, triglycerides, low-density lipoprotein cholesterol (LDL-C), high-density lipoprotein cholesterol (HDL-C), insulin, and high sensitivity C-reactive protein (hsCRP). Insulin resistance was assessed with the HOMA-IR as follows: fasting blood insulin (μU/mL) × fasting serum glucose (mmol/L)/22.5. The diagnosis of fatty liver was made based on abdominal ultrasound (US) by experienced radiologists who were blinded to the aim of the present study. Ultrasonographic diagnosis of fatty liver was defined as the presence of a diffuse increase of fine echoes in the liver parenchyma compared with the kidney or spleen parenchyma [32].

Being metabolically unhealthy was defined as having at least one of the following six metabolic abnormalities [33,34]: (1) fasting glucose level ≥100 mg/dL or current use of anti-diabetic medication; (2) BP ≥130/85 mmHg or current use of anti-hypertensives; (3) triglyceride levels ≥150 mg/dL or current use of lipid-lowering agents; (4) low HDL-C (<40 mg/dL in men or <50 mg/dL in women), (5) HOMA-IR score ≥2.5 [35]; or (6) fatty liver based on ultrasound [36]. Otherwise, being metabolically healthy was defined as having none of the metabolic abnormalities described above [33,37].

### 2.3. Statistical Analysis

We used a one-way ANOVA for continuous variables and a chi-squared test for categorical variables to compare the characteristics of participants at baseline by SRH. SRH was assessed in five categories; very good, good, fair, poor, and very poor. We combined the two lowest categories of poor and very poor because only 103 subjects were identified to be in the very poor category. Multiple comparisons were performed using Hochberg–Benjamini correction. 

The primary endpoints were development for each metabolic abnormality. Incidence density was expressed as the number of cases divided by person-years. Follow-up extended from the baseline exam until the development of each metabolic abnormality or the last health exam conducted before 31 December 2016.

We knew that metabolic abnormalities had occurred between two visits (visit with new-onset metabolic abnormality and the previous visit) but did not know the precise time of development. To account for this type of interval censoring, a parametric proportional hazard model was used to estimate hazard ratios and 95% confidence intervals for the association between SRH and metabolic abnormalities [38]. In these models, the baseline hazard function was parameterised with restricted cubic splines in log time with four degrees of freedom.

In order to control potential confounders, models were initially adjusted for age and sex, and then further adjusted for centre (Seoul or Suwon), year of screening exam, smoking status (never, past, current, or unknown), alcohol intake (0, <20, ≥20 g/day, or unknown), physical activity (inactive, minimally active, HEPA, or unknown), education level (high school graduate or less, community college or higher, and unknown), and total calorie intake (in quintile or missing), oral contraceptives, female sex hormone, male sex hormone, sleep duration (≤5, 6, 7, 8, or ≥9), CESD (<16, 16–<25, and ≥25) and BMI (continuous). To evaluate the impact of updated status of SRH and covariates over follow-up, we conducted additional analyses introducing SRH and other covariates as a time-varying covariate in the models.

We also evaluated whether or not the association between SRH and the development of metabolic abnormalities differs by the presence of obesity defined as a BMI ≥25 kg/m^2^ [29]. Additionally, we evaluated whether or not the association differs by the presence of abdominal obesity [30,31]. Interactions between SRH and obesity were tested using likelihood ratio tests comparing models with and without multiplicative interaction terms.

The statistical analysis was performed using STATA, version 14.0 (StataCorp LP, College Station, TX, USA). Two tailed *p* < 0.05 was considered statistically significant.

## 3. Results

Baseline characteristics of participants without metabolic abnormality at baseline are presented according to the presence of incident progression to metabolically unhealthy phenotype separately in non-obese and obese participants (Table 1). The proportion of participants transitioned from metabolically healthy phenotype into a metabolically unhealthy phenotype over follow-ups was 45.8% (36,178 out of 79,012) among non-obese individuals, whereas the corresponding proportion was 73.5% among obese individuals (4680 out of 6365). At baseline, individuals with progression to metabolically unhealthy phenotype were more likely to be older, male, current smokers, drink alcohol, and have high levels of BMI, BP, glucose, total cholesterol, LDL-C, triglycerides, ALT, and hsCRP in both non-obese and obese individuals. Appendix Table A1 shows age- and sex-adjusted mean values (95% CI) and proportion (95% CI) of baseline characteristics of participants by SRH categories. Poorer SRH was associated with younger age, female gender, current smoking, alcohol intake, lower physical activity, lower education level, and higher levels of total cholesterol, LDL-C, triglycerides, ALT, and HOMA-IR and were weakly associated with lower levels of BMI and HDL-C. Notably, poorer SRH was significantly associated with higher depressive symptoms.

Table 2 presents the development of each metabolic abnormality according to SRH category. During 258,689 person-years of follow-up, 40,858 participants developed any metabolic abnormality (incident rate, 157.9 per 1000 person-years) over a median follow-up period of 3.0 years (interquartile range, 1.9–4.1 years). Poorer SRH categories were associated with increased incidence of hypertriglyceridemia, high HOMA-IR, and fatty liver in a dose-response manner (*p* for trend < 0.05) (Table 2 and Appendix Table A2). In a fully-adjusted model including age, sex, centre, year of examination, smoking status, alcohol intake, physical activity, education level, total calorie intake, sleep duration, CESD, and BMI, HRs (95% CI) for incident hypertriglyceridemia comparing the “good”, “fair”, or “poor or very poor” vs. the “very good” self-rated health category were 1.04 (0.92–1.17), 1.16 (1.02–1.31), and 1.18 (1.02–1.36), respectively. Corresponding multivariable-adjusted HRs (95% CI) for high HOMA-IR were 1.17 (0.99–1.38), 1.42 (1.20–1.67), and 1.67 (1.39–2.00), respectively. Corresponding multivariable-adjusted HRs (95% CI) for incident fatty liver were 1.07 (0.95–1.21), 1.21 (1.07–1.37), and 1.26 (1.06–1.49), respectively. When updated status of SRH and confounders over time were treated as time-varying covariates, the association of SRH with an increased risk of high HOMA-IR and fatty liver remained significant (Table 2). There was no significant association between SRH and incidence of pre-diabetes, pre-hypertension, low HDL-C, and high hsCRP.

Table 3 and Table 4 show the association between SRH and development of metabolic abnormality in obese individuals and whether or not the association between SRH and development of each metabolic abnormality differs by obesity. The associations of SRH with the development of metabolic abnormalities were stronger in individuals with obesity than those without especially in relation to any metabolic abnormality (*p* for interaction = 0.039), fatty liver (*p* for interaction < 0.001), and high CRP (*p* for interaction = 0.030). In a fully-adjusted model, multivariable-adjusted HRs (95% CI) for any metabolic abnormality comparing the “good”, “fair”, or “poor or very poor” vs. the “very good” self-rated health category were 0.94 (0.89–1.01), 0.98 (0.92–1.04) and 0.97 (0.90–1.04), respectively, among non-obese subjects, whereas corresponding HRs (95% CI) among obese subjects were 1.05 (0.89–1.23), 1.13 (0.96–1.32), and 1.27 (1.04–1.54). Similarly, multivariable-adjusted HRs (95% CI) for incident fatty liver comparing the “good”, “fair”, or “poor or very poor” vs. the “very good” self-rated health category were 1.00 (0.87–1.15), 1.05 (0.92–1.21), and 1.02 (0.86–1.21), respectively, among non-obese subjects, whereas corresponding HRs (95% CI) among obese subjects were 1.16 (0.90–1.50), 1.46 (1.14–1.87), and 1.75 (1.30–2.35). The association between SRH and development of each metabolic abnormality did not differ by the presence of abdominal obesity (Appendix Table A3).

## 4. Discussion

In this large-scale cohort study of 85,377 metabolically healthy men and women, poorer SRH was significantly associated with a higher risk of developing hypertriglyceridemia, high HOMA-IR, and fatty liver in a dose-dependent manner. This association persisted even after adjustment for potential confounders, including demographic characteristics, depressive symptoms, and lifestyle factors. The association between SRH and progression to metabolically unhealthy status was much stronger in individuals with obesity than those without. Our findings suggest that SRH may be an independent risk factor of metabolic abnormalities, even in metabolically healthy individuals especially with obesity.

In this study, SRH was highly associated with depression. Similarly, in previous studies, depressive symptoms were significantly associated with poorer SRH [39,40]. A systematic review and meta-analysis of cross-sectional and cohort studies demonstrated a relationship between depression and metabolic syndrome [41]. Moreover, the association between depression and obesity has repeated been established in longitudinal studies and meta-analyses [42,43]. Given the interrelation between SRH, depression, and metabolic disorders, the higher risk of progression to metabolic unhealthy status in individuals with poorer SRH would be mediated by depression. However, in this study, after adjustment for potential confounders and depressive symptoms measured by CESD, poorer SRH was independently associated with development of metabolic abnormality. Thus, the association between SRH and metabolic abnormality could not be fully explained by depression.

Previous cross-sectional studies have shown that poorer SRH was associated with prevalence of metabolic abnormalities [44]. Our study findings are in line with results from previous studies and additionally demonstrated the prospective association of low SRH and the development of metabolic abnormalities. SRH appears to be related with physical health and lifestyle factors such as smoking, alcohol intake, physical activity, diet, and sleep duration. However, even after adjusting for those factors and depressive symptoms, the association between poor SRH and the incidence of hypertriglyceridemia, high HOMA-IR, and fatty liver persisted, indicating that the association of SRH with the development of metabolic abnormalities cannot be fully explained by health-related variables or depression. SRH is related to allostatic loads. Allostasis is the adaptive regulatory process that maintains homeostasis during exposure to stressors. The components of allostatic loads associated with SRH in previous studies include inflammatory biomarkers such as IL-6, IL-1, TNF-α, and CRP; endocrine factors such as cortisol, DHEA-S, IGF-1, testosterone, and oestradiol; and other biomarkers such as haemoglobin and white blood cell count [45]. The presence of social psychological resources such as social support and self-esteem was associated with better SRH in people with chronic disease and disability [46]. Given that SRH reflects many other factors that had not been explored, SRH would be a useful single indicator to predict health status such as metabolic health.

In our study, during a median follow-up of 3.0 years, the proportion of participants transitioned from metabolically healthy phenotype into a metabolically unhealthy phenotype over follow-ups was 45.8% (36,178 out of 79,012) among non-obese individuals, whereas the corresponding proportion was 73.5% among obese individuals (4680 out of 6365). Other previous studies have reported that the percentages of participants transitioning from metabolically healthy phenotype into a metabolically unhealthy state over follow-ups of 4–10 years ranged from 33 to 52% [47,48]. A recent study has reported that only a small proportion of women who were initially metabolically healthy obese stayed metabolically healthy (defined by absence of diabetes, hypertension and hypercholesterolemia) during follow-up (about 15% over 20 years) [48]. In the Tehran Lipid and Glucose Study which followed 916 metabolically healthy abdominal obese subjects for 10 years, nearly half of the metabolically healthy abdominal obese subjects lost their metabolic health and 42.1% developed metabolic syndrome [49]. Another study followed 85 Japanese Americans for 10 years showed that 64.7% of MHO participants converted to metabolically unhealthy obesity [50]. The different findings of conversion rate could be attributable to differences in study population (age, ethnicity and sex composition), definition of metabolic health and follow-up durations. Indeed, using stricter definition (e.g., zero metabolic abnormality) for metabolic health than using common definition such as metabolic syndrome, much larger proportion of participants over follow-up were classified as those who transitioned into metabolically unhealthy phenotype [51]. In our study, being metabolically healthy was defined as having none of the metabolic abnormalities including metabolic syndrome components, HOMA-IR, hsCRP and fatty liver [33,37]. Similarly, in the previous study, about 80% of MHO individuals became metabolically unhealthy during the average follow-up of 5.1 years (up to 7 years) [36]. 

Several mechanisms are suggested to explain the existence of metabolically healthy obesity (MHO) such as subclinical inflammation, expansion capacity of adipose tissue, circulating microRNA, and body composition [52]. The mechanism underlying transition from metabolically healthy phenotype to metabolically unhealthy phenotype is not fully understood. Individuals with metabolically healthy obesity might have favorable profiles of fat distribution such as more subcutaneous than visceral fat area and lower ectopic fat deposit than those with metabolically unhealthy obesity [10]. Adipose tissue is an active endocrine organ that produces and releases adipokines [53]. Adiponectin, one of the adipokines, is exclusively synthesized in adipose tissue and has insulin-sensitizing and anti-inflammatory properties, all of which are beneficial to metabolic health [54,55]. Studies have reported that adiponectin levels were significantly higher in metabolically healthy phenotype than in metabolically unhealthy phenotype [54], suggesting that adiponectin level may play an important role in determining the metabolic health. Further studies are needed to elucidate the mechanisms underlying the association between poorer SRH and conversion to metabolically unhealthy phenotype, especially in MHO individuals.

The impact of SRH on progression to metabolically unhealthy phenotype was greater in metabolically healthy obese people than non-obese people in this study. Previous studies have shown that SRH is low in people with metabolic syndrome [56,57,58] and obesity [59]. Even though obesity and increased BMI are important predictors of developing metabolic disorders in metabolically healthy individuals [36,60], no studies before our study evaluated an effect of obesity on an association between SRH and the development of metabolic abnormalities. In this study, the association of poorer SRH and the risk of fatty liver, high CRP, and any metabolic abnormality was stronger in obese individuals than in non-obese ones. The reason underlying the association between SRH and progression to metabolically unhealthy status especially in obese individuals is not fully understood. A pooled analysis of 30,337 men and women demonstrated that obese persons even with a favourable metabolic profile have a higher risk of depressive symptoms than those with normal weight [61]. 

Additionally, obesity has been reported to be associated with worse health-related quality of life regardless of metabolic status [62]. Thus, SRH assessed by a single question can reflect various aspects of health that are not commonly measured in a clinical setting or study and may be a simple tool to screen individuals at a high risk for metabolic disorders, especially in those with obesity. Further studies are required to better understand the difference in the association between SRH and metabolic abnormalities among obese individuals.

There are several limitations to this study. First, we used BMI as a measure of obesity, but BMI is limited by the lack of ability to distinguish fat tissue from lean tissue. If individuals with obesity had a higher proportion of lean mass compared to those with normal weight, the association between SRH and metabolic abnormalities in obese individuals could have been attenuated. Second, waist circumference measurements, an important indicator of visceral adiposity, were available only in a fraction of study participants; thus, we were unable to examine the role of fat distribution on the association between SRH and metabolic abnormalities. Third, information on specific types of working conditions was not available in our study; working at night, working on Sundays, and working more than 10 hours per day are also related with poor SRH [63]. This might have resulted in some degree of residual confounding. Lastly, our study was conducted on highly educated, young and middle-aged Koreans who regularly participate in health checkup exams; thus, our findings might not be generalizable to other age groups, populations with a higher prevalence of comorbidities or different characteristics, or to other racial/ethnic groups.

Despite the above limitations, our study also has several strengths including the large sample size and the use of carefully standardised clinical, imaging, and laboratory procedures. Second, our findings are from metabolically healthy and relatively younger participants who are less likely to be affected by comorbidities that older cohorts. Except for a study in adolescents [45], this is the first study to demonstrate a role of SRH in the development of metabolic abnormalities in a large cohort.

## 5. Conclusions

The risk of progression to metabolically unhealthy status was higher in individuals with poorer SRH, especially in those with obesity. Our findings suggest SRH as an independent risk factor for progression to metabolically unhealthy status especially among metabolically healthy obese individuals. SRH may help identify metabolically healthy obese individuals at high risk for progression to a metabolically unhealthy phenotype.

## Figures and Tables

**Table 1:** Baseline characteristics of study participants according to progression to metabolic unhealthy status by obesity.

Characteristics	Non-Obese Participants		*p*-Value	Obese Participants		*p*-Value
	No Progression to Metabolic Unhealthy	Progression to Metabolic Unhealthy		No Progression to Metabolic Unhealthy	Progression to Metabolic Unhealthy	
Number	42,834	36,178		1,685	4,680	
Age (years) ^a^	35.3 (6.1)	36.6 (6.3)	<0.001	35.6 (6.6)	36.8 (6.6)	<0.001
Male (%)	23.6	43.5	<0.001	62.3	73.3	<0.001
Current smoker (%)	11.6	22.6	<0.001	26.8	35.8	<0.001
Alcohol intake (%) ^b^	10.2	16.7	<0.001	22.5	29.0	<0.001
HEPA (%)	14.5	15.8	<0.001	22.0	21.9	0.890
High education level (%) ^c^	85.2	84.2	0.001	85.7	84.6	0.326
BMI (kg/m^2^)	20.5 (2.0)	21.4 (2.0)	<0.001	26.2 (1.1)	26.4 (1.3)	<0.001
Systolic BP (mmHg) ^a^	100.6 (9.2)	104.4 (10.1)	<0.001	108.2 (8.9)	110.5 (8.8)	<0.001
Diastolic BP (mmHg) ^a^	64.0 (7.0)	66.5 (7.5)	<0.001	67.0 (6.9)	69.2 (7.1)	<0.001
Glucose (mg/dL) ^a^	88.2 (6.0)	90.1 (5.8)	<0.001	89.7 (5.7)	91.2 (5.3)	<0.001
Total cholesterol (mg/dL) ^a^	182.5 (28.6)	187.8 (29.8)	<0.001	189.4 (28.9)	195.3 (30.5)	<0.001
LDL-C (mg/dL) ^d^	104.1 (25.8)	112.6 (28.1)	<0.001	117.1 (26.4)	124.6 (28.9)	<0.001
HDL-C (mg/dL) ^d^	69.5 (13.2)	64.1 (13.0)	<0.001	62.2 (12.0)	57.9 (11.3)	<0.001
Triglycerides (mg/dL) ^d^	63 (50–80)	73 (57–95)	<0.001	76 (59–98)	87 (67–110)	<0.001
HOMA-IR ^d^	0.85 (0.58–1.19)	0.89 (0.60–1.24)	<0.001	1.07 (0.75–1.47)	1.13 (0.80–1.54)	<0.001
hsCRP (mg/L) ^d^	0.2 (0.2–0.4)	0.3 (0.2–0.5)	<0.001	0.3 (0.2–0.5)	0.4 (0.3–0.6)	<0.001
Total energy intake (kcal/day) ^d,^^e^	1445.4 (1090.3–1833.2)	1552.0 (1199.2–1935.2)	<0.001	1569.8 (1203.2–1967.3)	1664.3 (1291.0–2099.1)	<0.001
Sleep duration (h)	6.7 (1.2)	6.6 (1.1)	<0.001	6.4 (1.1)	6.4 (1.1)	0.462
CES-D ≥ 16 (%)	14.0	12.2	<0.001	11.6	10.5	0.265

Data are ^a^ means (standard deviation), or percentages. ^b^ ≥20 g of ethanol per day; ^c^ ≥College graduate. Data are ^d^ medians (interquartile range). ^e^ among 54,257 participants with plausible estimated energy intake levels (within three standard deviations of the log-transformed mean energy intake). BMI: body mass index, BP: blood pressure, CES-D: the Centre for Epidemiologic Studies Depression Scale, CI: confidence interval, HDL-C: high-density lipoprotein-cholesterol, HEPA: health-enhancing physical activity, hsCRP: high-sensitivity C-reactive protein, HOMA-IR: homeostasis model assessment of insulin resistance.

**Table 2:** Development of each metabolic abnormality by self-rated health category.

Self-Rated Health Category	Person-Years	Incident Cases	Incidence Density (per 1000 Person-Years)	Age and Sex-Adjusted HR (95% CI)	Multivariate HR ^a^ (95% CI)	HR (95% CI) ^b^ in Model Using Time-Dependent Variables
Any metabolic abnormality						
Very good	8072.1	1380	171.0	1.00 (reference)	1.00 (reference)	1.00 (reference)
Good	81,969.6	13,201	161.0	0.96 (0.91–1.02)	0.96 (0.92–1.04)	0.98 (0.92–1.04)
Fair	150,743.4	23,537	156.1	1.01 (0.95–1.06)	1.04 (0.98–1.10)	1.00 (0.94–1.06)
Bad or very bad	17,904.0	2740	153.0	1.04 (0.97–1.11)	1.07 (0.99–1.15)	1.01 (0.94–1.08)
*p* for trend				<0.001	<0.001	0.119
Hypertriglyceridemia						
Very good	9799.1	329	33.6	1.00 (reference)	1.00 (reference)	1.00 (reference)
Good	99,796.1	3342	33.5	1.05 (0.93–1.17)	1.04 (0.92–1.17)	0.99 (0.88–1.11)
Fair	181,754.8	5864	32.3	1.16 (1.04–1.30)	1.16 (1.02–1.31)	1.08 (0.96–1.21)
Bad or very bad	21,402.4	666	31.1	1.22 (1.07–1.40)	1.18 (1.02–1.36)	1.12 (0.98–1.29)
*p* for trend				<0.001	<0.001	<0.001
Pre-diabetes						
Very good	9555.5	471	49.3	1.00 (reference)	1.00 (reference)	1.00 (reference)
Good	97,698.2	4401	45.0	0.95 (0.86–1.04)	0.99 (0.89–1.10)	1.07 (0.96–1.19)
Fair	178,411.5	7504	42.1	0.97 (0.89–1.07)	1.00 (0.90–1.11)	1.05 (0.94–1.16)
Bad or very bad	21,004.4	823	39.2	0.98 (0.87–1.10)	1.00 (0.88–1.13)	1.03 (0.91–1.16)
*p* for trend				0.429	0.697	0.512
Pre-hypertension						
Very good	10,017.0	181	18.1	1.00 (reference)	1.00 (reference)	1.00 (reference)
Good	102,517.7	1632	15.9	0.95 (0.81–1.10)	0.99 (0.84–1.17)	0.98 (0.83–1.16)
Fair	186,463.3	2663	14.3	0.99 (0.86–1.16)	1.05 (0.89–1.24)	1.00 (0.85–1.18)
Bad or very bad	21,899.4	277	12.6	0.99 (0.82–1.19)	1.01 (0.82–1.25)	1.02 (0.84–1.24)
*p* for trend				0.286	0.235	0.550
Low HDL-C						
Very good	10,061.8	183	18.2	1.00 (reference)	1.00 (reference)	1.00 (reference)
Good	101,581.6	1972	19.4	1.04 (0.89–1.21)	1.04 (0.88–1.23)	1.01 (0.86–1.19)
Fair	184,102.6	3745	20.3	1.07 (0.92–1.25)	1.13 (0.96–1.34)	1.05 (0.90–1.24)
Bad or very bad	21,576.1	444	20.6	1.09 (0.92–1.30)	1.10 (0.90–1.33)	1.05 (0.88–1.27)
*p* for trend				0.141	0.019	0.186
High HOMA-IR						
Very good	10,094.7	185	18.3	1.00 (reference)	1.00 (reference)	1.00 (reference)
Good	102,359.0	2128	20.8	1.09 (0.94–1.27)	1.17 (0.99–1.38)	1.23 (1.04–1.45)
Fair	184,998.9	4483	24.2	1.32 (1.14–1.53)	1.42 (1.21–1.67)	1.39 (1.19–1.63)
Bad or very bad	21,664.1	611	28.2	1.55 (1.31–1.83)	1.67 (1.39–2.00)	1.57 (1.31–1.88)
*p* for trend				<0.001	<0.001	<0.001
Fatty liver						
Very good	9830.4	339	34.5	1.00 (reference)	1.00 (reference)	1.00 (reference)
Good	99,779.8	3274	32.8	1.01 (0.90–1.13)	1.07 (0.95–1.21)	1.07 (0.95–1.21)
Fair	181,999.0	5504	30.2	1.08 (0.97–1.21)	1.21 (1.07–1.37)	1.13 (1.01–1.28)
Bad or very bad	21,258.5	634	30.0	1.17 (1.03–1.34)	1.28 (1.10–1.48)	1.22 (1.05–1.40)
*p* for trend				<0.001	<0.001	<0.001
High CRP						
Very good	9453.9	618	65.4	1.00 (reference)	1.00 (reference)	1.00 (reference)
Good	95,214.0	6070	63.8	0.98 (0.90–1.06)	0.98 (0.89–1.07)	0.97 (0.89–1.06)
Fair	173,662.0	10,831	62.4	1.00 (0.92–1.09)	1.01 (0.92–1.10)	0.96 (0.88–1.04)
Bad or very bad	20,371.9	1253	61.5	1.02 (0.93–1.12)	1.04 (0.94–1.16)	0.98 (0.88–1.08)
*p* for trend				0.120	0.060	0.436

^a^ Estimated from parametric proportional hazard models. Multivariable model was adjusted for age, sex, centre, year of screening exam, smoking status, alcohol intake, physical activity, education level, total calorie intake, oral contraceptives, hormone replacement therapy, sleep duration, CESD and BMI. ^b^ Estimated from parametric proportional hazard models with self-rated health category, smoking status, alcohol intake, physical activity, BMI, total calorie intake, oral contraceptives, hormone replacement therapy, testosterone, sleep duration, CESD categories as time-dependent variables and baseline age, sex, center, year of screening exam and education level as time-fixed variables. *BMI*: body mass index, CESD: the Centre for Epidemiologic Studies Depression Scale, CI: confidence interval, HDL-C: high-density lipoprotein-cholesterol, HOMA-IR: homeostasis model assessment of insulin resistance, HR: hazard ratios, hsCRP: high-sensitivity C-reactive protein.

**Table 3:** Development of each metabolic abnormality by self-rated health category among metabolic healthy obese participants.

Self-Rated Health Category	Person-Years	Incident Cases	Incidence Density (per 1000 Person-Years)	Age and Sex-Adjusted HR (95% CI)	Multivariate HR ^a^ (95% CI)	HR (95% CI) ^b^ in Model Using Time-Dependent Variables
Any metabolic abnormality						
Very good	949.3	230	242.3	1.00 (reference)	1.00 (reference)	1.00 (reference)
Good	7729.5	1897	245.4	1.05 (0.92–1.21)	1.05 (0.89–1.23)	1.05 (0.94–1.18)
Fair	11,586.0	2910	251.2	1.16 (1.01–1.33)	1.13 (0.96–1.32)	1.07 (0.96–1.20)
Bad or very bad	1360.5	359	263.9	1.29 (1.09–1.52)	1.27 (1.04–1.54)	1.08 (0.95–1.24)
*p* for trend				<0.001	0.005	0.188
Hypertriglyceridemia						
Very good	1246.7	65	52.1	1.00 (reference)	1.00 (reference)	1.00 (reference)
Good	10,187.1	604	59.3	1.20 (0.93–1.55)	1.24 (0.92–1.66)	1.05 (0.86–1.28)
Fair	15,090.5	898	59.5	1.36 (1.06–1.75)	1.33 (0.99–1.78)	1.13 (0.93–1.37)
Bad or very bad	1774.1	112	63.1	1.60 (1.18–2.17)	1.51 (1.06–2.14)	1.20 (0.95–1.50)
*p* for trend				<0.001	0.032	0.020
Pre-diabetes						
Very good	1239.1	77	62.1	1.00 (reference)	1.00 (reference)	1.00 (reference)
Good	9978.8	687	68.8	1.19 (0.94–1.51)	1.26 (0.95–1.67)	1.12 (0.93–1.36)
Fair	14,883.8	1001	67.3	1.27 (1.01–1.61)	1.31 (0.99–1.73)	1.08 (0.89–1.30)
Bad or very bad	1750.7	125	71.4	1.43 (1.07–1.90)	0.40 (1.00–1.96)	1.09 (0.87–1.36)
*p* for trend				0.007	0.192	0.804
Pre-hypertension						
Very good	1245.2	51	41.0	1.00 (reference)	1.00 (reference)	1.00 (reference)
Good	10,615.8	343	32.3	0.83 (0.61–1.11)	0.88 (0.63–1.24)	0.96 (0.74–1.25)
Fair	15,693.7	510	32.5	0.95 (0.71–1.26)	0.95 (0.68–1.33)	1.02 (0.78–1.32)
Bad or very bad	1868.5	67	35.9	1.13 (0.79–1.63)	1.19 (0.79–1.81)	1.25 (0.92–1.70)
*p* for trend				0.056	0.202	0.047
Low HDL-C						
Very good	1312.9	22	16.8	1.00 (reference)	1.00 (reference)	1.00 (reference)
Good	10,618.1	288	27.1	1.57 (1.02–2.43)	1.45 (0.90–2.35)	1.09 (0.81–1.46)
Fair	15,705.1	461	29.4	1.65 (1.07–2.53)	1.50 (0.93–2.41)	1.15 (0.85–1.53)
Bad or very bad	1853.0	62	33.5	1.84 (1.13–3.00)	1.59 (0.92–2.75)	1.18 (0.84–1.65)
*p* for trend				0.036	0.261	0.199
High HOMA-IR						
Very good	1281.2	50	39.0	1.00 (reference)	1.00 (reference)	1.00 (reference)
Good	10,484.4	473	45.1	1.10 (0.82–1.48)	0.96 (0.70–1.32)	1.14 (0.89–1.44)
Fair	15,336.6	875	57.1	1.42 (1.06–1.88)	1.19 (0.88–1.62)	1.28 (1.02–1.62)
Bad or very bad	1795.7	129	71.8	1.78 (1.28–2.47)	1.43 (0.99–2.04)	1.41 (1.08–1.83)
*p* for trend				<0.001	0.001	<0.001
Fatty liver						
Very good	1201.0	98	81.6	1.00 (reference)	1.00 (reference)	1.00 (reference)
Good	9855.8	805	81.7	1.01 (0.82–1.25)	1.16 (0.90–1.50)	1.121 (0.94–1.33)
Fair	14,468.2	1302	90.0	1.22 (0.99–1.50)	1.46 (1.14–1.87)	1.17 (0.99–1.40)
Bad or very bad	1678.8	175	104.2	1.49 (1.16–1.91)	1.75 (1.30–2.35)	1.32 (1.07–1.61)
*p* for trend				<0.001	<0.001	0.003
High CRP						
Very good	1231.2	95	77.2	1.00 (reference)	1.00 (reference)	1.00 (reference)
Good	9716.1	844	86.9	1.14 (0.92–1.41)	1.23 (0.95–1.58)	1.10 (0.93–1.31)
Fair	14,430.7	1340	92.9	1.25 (1.01–1.54)	1.31 (1.02–1.68)	1.06 (0.90–1.25)
Bad or very bad	1688.2	158	93.6	1.28 (0.99–1.66)	1.47 (1.09–1.99)	1.13 (0.93–1.37)
*p* for trend				0.005	0.106	0.907

^a^ Estimated from parametric proportional hazard models. Multivariable model was adjusted for age, sex, centre, year of screening exam, smoking status, alcohol intake, physical activity, education level, total calorie intake, oral contraceptives, hormone replacement therapy, testosterone, sleep duration, and CESD. ^b^ Estimated from parametric proportional hazard models with self-rated health category, smoking status, alcohol intake, physical activity, total calorie intake, oral contraceptives, hormone replacement therapy, male sex hormone, sleep duration, CESD categories as time-dependent variables and baseline age, sex, center, year of screening exam and education level as time-fixed variables. BMI: body mass index, CESD: the Centre for Epidemiologic Studies Depression Scale, CI: confidence interval, HDL-C: high-density lipoprotein-cholesterol, HOMA-IR: homeostasis model assessment of insulin resistance, HR: hazard ratios, hsCRP: high-sensitivity C-reactive protein.

**Table 4:** Hazard ratios (95% CI) of development of each metabolic abnormality according to self-rated health category and obesity.

Subgroup	Self-Rated Health Category				*p* for Trend	*p* for Interaction
	Very Good	Good	Fair	Bad or Very Bad		
Any metabolic abnormality						0.039
<25 kg/m^2^ (*n* = 79,012)	reference	0.94 (0.89–1.01)	0.98 (0.92–1.04)	0.97 (0.90–1.04)	0.151	
≥25 kg/m^2^ (*n* = 6365)	reference	1.05 (0.89–1.23)	1.13 (0.96–1.32)	1.27 (1.04–1.54)	0.005	
Hypertriglyceridemia						0.300
<25 kg/m^2^ (*n* = 79,012)	reference	0.98 (0.86–1.12)	1.07 (0.94–1.23)	1.04 (0.89–1.23)	0.005	
≥25 kg/m^2^ (*n* = 6365)	reference	1.24 (0.92–1.66)	1.33 (0.99–1.78)	1.51 (1.06–2.14)	0.032	
Pre-diabetes						0.080
<25 kg/m^2^ (*n* = 79,012)	reference	0.93 (0.83–1.04)	0.92 (0.83–1.03)	0.89 (0.78–1.02)	0.195	
≥25 kg/m^2^ (*n* = 6365)	reference	1.26 (0.95–1.67)	1.31 (0.99–1.73)	1.40 (1.00–1.96)	0.192	
Pre-hypertension						0.094
<25 kg/m^2^ (*n* = 79,012)	reference	1.00 (0.83–1.22)	1.03 (0.85–1.24)	0.89 (0.70–1.13)	0.722	
≥25 kg/m^2^ (*n* = 6365)	reference	0.88 (0.63–1.24)	0.95 (0.68–1.33)	1.19 (0.79–1.81)	0.202	
Low HDL-C						0.384
<25 kg/m^2^ (*n* = 79,012)	reference	0.96 (0.80–1.15)	1.03 (0.86–1.23)	0.96 (0.78–1.18)	0.337	
≥25 kg/m^2^ (*n* = 6365)	reference	1.45 (0.90–2.35)	1.50 (0.93–2.41)	1.59 (0.92–2.75)	0.261	
High HOMA-IR						0.528
<25 kg/m^2^ (*n* = 79,012)	reference	1.20 (0.99–1.46)	1.40 (1.16–1.70)	1.58 (1.28–1.95)	<0.001	
≥25 kg/m^2^ (*n* = 6365)	reference	0.96 (0.70–1.32)	1.19 (0.88–1.62)	1.43 (0.99–2.04)	0.001	
Fatty liver						<0.001
<25 kg/m^2^ (*n* = 79,012)	reference	1.00 (0.87–1.15)	1.05 (0.92–1.21)	1.02 (0.86–1.21)	0.037	
≥25 kg/m^2^ (*n* = 6365)	reference	1.16 (0.90–1.50)	1.46 (1.14–1.87)	1.75 (1.30–2.35)	<0.001	
High CRP						0.030
<25 kg/m^2^ (*n* = 79,012)	reference	0.93 (0.84–1.02)	0.93 (0.85–1.03)	0.93 (0.83–1.05)	0.909	
≥25 kg/m^2^ (*n* = 6365)	reference	1.23 (0.95–1.58)	1.31 (1.02–1.68)	1.47 (1.09–1.99)	0.106	

Estimated from parametric proportional hazard models adjusted for age, sex, centre, year of screening exam, smoking status, alcohol intake, total calorie intake, oral contraceptives, hormone replacement therapy, male sex hormone, sleep duration, CESD, physical activity, and education. BMI: body mass index, CESD: the Centre for Epidemiologic Studies Depression Scale, CI: confidence interval, HDL-C: high-density lipoprotein-cholesterol, HOMA-IR: homeostasis model assessment of insulin resistance, HR: hazard ratios, hsCRP: high-sensitivity C-reactive protein.

## Appendix A

**Table A1:** Estimated ^a^ mean values (95% CI) and adjusted ^a^ proportion (95% CI) of baseline characteristics of study participants by self-rated health.

Characteristics	Self-Rated Health Category				*p* for Trend	Multiple Comparison ^g^
	Very Good (a)	Good (b)	Fair (c)	Poor or Very Poor (d)		
Number	2825	27,048	49,626	5878		
Age (years) ^b^	36.2 (36.0–36.4)	36.0 (35.9–36.1)	35.9 (35.9–36.0)	35.3 (35.2–35.5)	<0.001	a ≠ d, b ≠ d, c ≠ d
Male (%)	49.7 (47.8–51.5)	42.4 (41.8–43.0)	31.9 (31.5–32.3)	27.1 (25.9–28.2)	<0.001	a ≠ b ≠ c ≠ d
Current smoker (%)	13.6 (12.5–14.6)	16.4 (16.0–16.8)	18.8 (18.5–19.2)	21.6 (20.6–22.7)	<0.001	a ≠ b ≠ c ≠ d
Alcohol intake (%) ^c^	13.4 (12.3–14.5)	13.6 (13.2–13.9)	14.4 (14.1–14.7)	17.2 (16.2–18.2)	<0.001	a ≠ d, b ≠ c ≠ d
HEPA (%)	30.7 (29.0–32.4)	20.0 (19.5–20.5)	12.9 (12.6–13.2)	10.6 (9.8–11.4)	<0.001	a ≠ b ≠ c ≠ d
High education level (%) ^d^	85.7 (84.2–87.1)	86.2 (85.8–86.7)	84.4 (84.1–84.8)	81.2 (80.1–82.2)	<0.001	a ≠ d, b ≠ c ≠ d
Obesity	8.1 (7.2–9.0)	7.4 (7.1–7.7)	7.3 (7.1–7.6)	8.1 (7.4–8.8)	0.872	-
BMI (kg/m^2^)	21.7 (21.6–21.8)	21.5 (21.4–21.5)	21.3 (21.2–21.3)	21.1 (21.0–21.1)	<0.001	a ≠ b ≠ c ≠ d
Systolic BP (mmHg) ^b^	103.2 (102.9–103.5)	103.1 (103.0–103.2)	102.8 (102.8–102.9)	102.1 (101.9–102.4)	<0.001	a ≠ d, b ≠ c ≠ d
Diastolic BP (mmHg) ^b^	65.0 (64.8–65.3)	65.3 (65.2–65.4)	65.5 (65.4–65.5)	65.3 (65.1–65.5)	0.001	a ≠ c, b ≠ c
Glucose (mg/dL) ^b^	89.1 (88.9–89.3)	89.1 (89.1–89.2)	89.3 (89.2–89.3)	89.1 (89.0–89.3)	0.026	b ≠ c
Total cholesterol (mg/dL) ^b^	183.3 (182.2–184.4)	185.2 (184.9–185.6)	185.9 (185.6–186.1)	185.5 (184.8–186.3)	<0.001	a ≠ b ≠ c, a ≠ d
LDL-C (mg/dL) ^b^	106.3 (105.4–107.3)	108.5 (108.2–108.8)	109.6 (109.3–109.8)	109.5 (108.8–110.1)	<0.001	a ≠ b ≠ c, a ≠ d, b ≠ d
HDL-C (mg/dL) ^b^	67.7 (67.3–68.2)	66.9 (66.7–67.0)	66.2 (66.1–66.3)	65.8 (65.5–66.1)	<0.001	a ≠ b ≠ c ≠ d
Triglycerides (mg/dL) ^e^	69.4 (68.5–70.3)	71.9 (71.6–72.2)	73.7 (73.5–74.0)	74.4 (73.8–75.0)	<0.001	a ≠ b ≠ c ≠ d
HOMA-IR ^e^	0.92 (0.90–0.93)	0.94 (0.93–0.94)	0.97 (0.96–0.97)	0.96 (0.95–0.97)	<0.001	a ≠ b ≠ c, a ≠ d, b ≠ d
hsCRP (mg/L) ^e^	0.34 (0.33–0.34)	0.33 (0.33–0.33)	0.33 (0.32–0.33)	0.32 (0.31–0.32)	<0.001	a ≠ b ≠ c ≠ d
Total energy intake (kcal/day) ^e,f^	1575.3 (1547.4–1603.1)	1575.3 (1566.5–1584.2)	1559.0 (1552.5–1565.5)	1569.0 (1550.2–1587.8)	0.058	b ≠ c
Sleep duration (h)	6.7 (6.6–6.7)	6.6 (6.6–6.7)	6.6 (6.6–6.6)	6.5 (6.4–6.5)	<0.001	a ≠ c, a ≠ d, b ≠ c≠ d
CES-D ≥ 16 (%)	4.6 (3.7–5.4)	7.3 (6.9–7.6)	13.9 (13.6–14.2)	33.4 (32.2–34.7)	<0.001	a ≠ b ≠ c ≠ d

^a^ Adjusted for age and sex; Data are ^b^ means (standard deviation), ^e^ medians (interquartile range), or percentages. BMI: body mass index, BP: blood pressure, CES-D: the Centre for Epidemiologic Studies Depression Scale, CI: confidence interval, HDL-C: high-density lipoprotein-cholesterol, HEPA: health-enhancing physical activity, hsCRP: high-sensitivity C-reactive protein, HOMA-IR: homeostasis model assessment of insulin resistance. ^c^ ≥20 g of ethanol per day; ^d^ ≥college graduate; ^f^ among 54,257 participants with plausible estimated energy intake levels (within three standard deviations of the log-transformed mean energy intake); ^g^ multiple comparison analysis was performed with Hochberg-Benjamini correction.

**Table A2:** Hazard ratios (95% CI) ^a^ for each metabolic abnormality by self-rated health category with multiple comparison.

Each Metabolic Abnormality	Self-Rated Health Category				*p* for Trend	Multiple Comparison ^b^
	Very Good (a)	Good (b)	Fair (c)	Bad or Very Bad (d)		
Any metabolic abnormality	1.00 (reference)	0.96 (0.92–1.04)	1.04 (0.98–1.10)	1.07 (0.99–1.15)	<0.001	b ≠ c, b ≠ d
Hypertriglyceridemia	1.00 (reference)	1.04 (0.92–1.17)	1.16 (1.02–1.31)	1.18 (1.02–1.36)	<0.001	a ≠ c, a ≠ d, b ≠ c ≠ d
Pre-diabetes	1.00 (reference)	0.99 (0.89–1.10)	1.00 (0.90–1.11)	1.00 (0.88–1.13)	0.697	-
Pre-hypertension	1.00 (reference)	0.99 (0.84–1.17)	1.05 (0.89–1.24)	1.01 (0.82–1.25)	0.235	-
Low HDL-C	1.00 (reference)	1.04 (0.88–1.23)	1.13 (0.96–1.34)	1.10 (0.90–1.33)	0.019	b ≠ c
High HOMA-IR	1.00 (reference)	1.17 (0.99–1.38)	1.42 (1.21–1.67)	1.67 (1.39–2.00)	<0.001	a ≠ c, a ≠ d, b ≠ c ≠ d
Fatty liver	1.00 (reference)	1.07 (0.95–1.21)	1.21 (1.07–1.37)	1.28 (1.10–1.48)	<0.001	a ≠ c, a ≠ d, b ≠ c, b ≠ d
High CRP	1.00 (reference)	0.98 (0.89–1.07)	1.01 (0.92–1.10)	1.04 (0.94–1.16)	0.06	-

^a^ Estimated from parametric proportional hazard models. Multivariable model was adjusted for age, sex, centre, year of screening exam, smoking status, alcohol intake, physical activity, education level, total calorie intake, oral contraceptives, hormone replacement therapy, testosterone, sleep duration, CESD and BMI. ^b^ Multiple comparison analysis was performed with Hochberg-Benjamini correction.

**Table A3:** Hazard ratios (95% CI) ^a^ of development of each metabolic abnormality according to self-rated health category stratified by abdominal obesity (*n* = 73,616).

Subgroup	Self-Rated Health Category				*p* for Trend	*p* for Interaction
	Very Good	Good	Fair	Bad or Very Bad		
Any metabolic abnormality						0.945
No abdominal obesity (*n* = 65,937)	reference	0.92 (0.85–0.98)	0.95 (0.88–1.02)	0.96 (0.88–1.04)	0.188	
Abdominal obesity (*n* = 7679)	reference	0.93 (0.80–1.08)	0.98 (0.85–1.13)	0.99 (0.82–1.18)	0.195	
Hypertriglyceridemia						0.734
No abdominal obesity (*n* = 65,937)	reference	1.04 (0.89–1.21)	1.11 (0.96–1.30)	1.09 (0.91–1.31)	0.046	
Abdominal obesity (*n* = 7679)	reference	1.01 (0.78–1.31)	1.12 (0.87–1.45)	1.21 (0.89–1.66)	0.015	
Pre-diabetes						0.822
No abdominal obesity (*n* = 65,937)	reference	0.96 (0.84–1.09)	0.96 (0.84–1.09)	0.95 (0.81–1.10)	0.609	
Abdominal obesity (*n* = 7679)	reference	0.93 (0.73–1.18)	0.98 (0.78–1.24)	0.95 (0.70–1.28)	0.627	
Pre-hypertension						0.484
No abdominal obesity (*n* = 65,937)	reference	1.07 (0.86–1.34)	1.04 (0.84–1.30)	1.01 (0.77–1.32)	0.558	
Abdominal obesity (*n* = 7679)	reference	0.86 (0.63–1.18)	0.94 (0.69–1.28)	0.91 (0.61–1.36)	0.519	
Low HDL-C						0.272
No abdominal obesity (*n* = 65,937)	reference	0.90 (0.74–1.10)	0.98 (0.81–1.19)	0.99 (0.79–1.24)	0.124	
Abdominal obesity (*n* = 7679)	reference	1.15 (0.73–1.82)	1.26 (0.80–1.98)	0.88 (0.49–1.57)	0.501	
High HOMA-IR						0.520
No abdominal obesity (*n* = 65,937)	reference	1.18 (0.95–1.46)	1.36 (1.10–1.68)	1.56 (1.23–1.96)	<0.001	
Abdominal obesity (*n* = 7679)	reference	0.95 (0.69–1.31)	1.18 (0.86–1.62)	1.45 (1.00–2.10)	<0.001	
Fatty liver						0.411
No abdominal obesity (*n* = 65,937)	reference	0.90 (0.77–1.05)	1.00 (0.86–1.16)	1.02 (0.85–1.23)	0.005	
Abdominal obesity (*n* = 7679)	reference	1.14 (0.90–1.45)	1.24 (0.98–1.57)	1.34 (1.01–1.79)	0.069	
High CRP						0.232
No abdominal obesity (*n* = 65,937)	reference	0.92 (0.83–1.02)	0.92 (0.83–1.02)	0.94 (0.83–1.07)	0.796	
Abdominal obesity (*n* = 7679)	reference	1.01 (0.80–1.28)	1.10 (0.88–1.39)	1.09 (0.82–1.45)	0.234	

^a^ Estimated from parametric proportional hazard models adjusted for age, sex, centre, year of screening exam, smoking status, alcohol intake, total calorie intake, oral contraceptives, hormone replacement therapy, male sex hormone, sleep duration, CESD, physical activity, and education. BMI: body mass index, CESD: the Centre for Epidemiologic Studies Depression Scale, CI: confidence interval, HDL-C: high-density lipoprotein-cholesterol, HOMA-IR: homeostasis model assessment of insulin resistance, HR: hazard ratios, hsCRP: high-sensitivity C-reactive protein.
